# Facile synthesis of polyethylenimine coated Bi_2_O_3_/Gd_2_O_3_ composite nanoparticles as multimodal MRI/CT contrast agents

**DOI:** 10.1039/d5ra07455j

**Published:** 2025-12-08

**Authors:** Le T. T. Tam, Le V. Thanh, Le G. Nam, Doan T. Tung, Hoang T. Dung, Le T. Tam, Ho D. Quang, Ngo T. Dung, Le T. Lu

**Affiliations:** a Institute of Materials Science, Vietnam Academy of Science and Technology 18 Hoang Quoc Viet Hanoi Vietnam lult@ims.vast.ac.vn; b Graduate University of Science and Technology, Vietnam Academy of Science and Technology 18 Hoang Quoc Viet Hanoi Vietnam; c Hoan My Vinh Hospital 99 Pham Đ inh Toai Vinh Phu Ward Nghe An Province Vietnam; d Vinh University 182 Le Duan, Truong Vinh Ward Nghe An Province Vietnam

## Abstract

Bimodal imaging contrast agents integrated magnetic resonance imaging (MRI) and computed tomography (CT) have attracted significant attention in recent years. In this study, we report the design and synthesis of Bi_2_O_3_/Gd_2_O_3_@polyethylenimine composite nanoparticles (BGO@PEI NPs) by a facile wet chemistry method. The BGO@PEI NPs exhibit uniform spherical morphology with an average core diameter of around 5.8 nm, high colloidal stability, and excellent biocompatibility. Comprehensive characterization confirmed their efficient *r*_1_ relaxation enhancement and strong X-ray attenuation. The BGO@PEI NPs revealed a superior MRI *T*_1_ contrast performance with a longitudinal relaxivity (*r*_1_) of 16.52 mM^−1^ s^−1^, exceeding that of clinical MRI agents, while also exhibited a CT attenuation coefficient of 16.59 HU mM^−1^ at 80 kV. *In vivo* imaging confirmed significant tumor accumulation and dual MRI/CT signal enhancement, with CT intensity increasing from 21.5 to 80.4 HU and *T*_1_-weighted ΔSNR rising by 75% within 60 min post-injection. These results demonstrate that BGO@PEI NPs represent a promising multifunctional platform for dual-modal MRI/CT imaging and potential applications in precision diagnostics.

## Abstract 2

Bi_2_O_3_/Gd_2_O_3_@PEI composite nanoparticles synthesized *via* a simple one-pot method exhibit high stability, biocompatibility, and outstanding dual-modal CT/MRI imaging performance.

## Graphical abstract

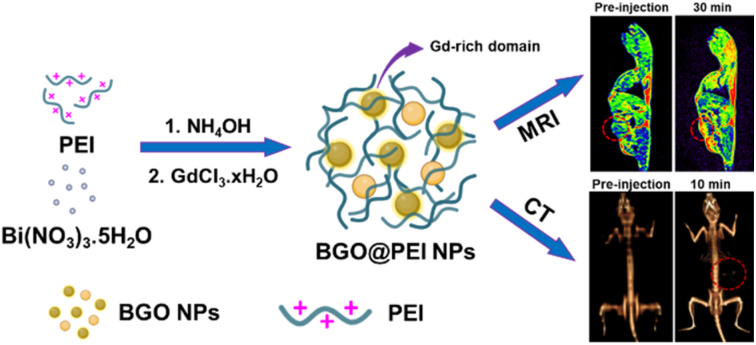

## Introduction

MRI and CT are among the most widely employed medical imaging techniques, each offering unique diagnostic insights with specific advantages and limitations.^1,2^ MRI is highly effective for visualizing detailed images of soft tissues due to its superior spatial resolution.^3,4^ However, it suffers from low sensitivity and relatively long acquisition time.^5^ In contrast, CT offers rapid imaging with high spatial resolution, making it indispensable for detecting bone abnormalities, vascular lesions, and acute injuries.^6,7^ Nevertheless, CT suffers from poor soft tissue contrast and involves exposure to significant radiation.^8^ To overcome these drawbacks, increasing efforts have focused on the development of bimodal or multimodal contrast agents that combine the strengths of both MRI and CT in a single nanoplatform.^9,10^

Clinically approved MRI contrast agents are predominantly gadolinium (Gd)-based chelates, which rely on the strong paramagnetism of Gd^3+^ ions.^11,12^ However, their low longitudinal relaxivity (*r*_1_), rapid renal clearance, and potential toxicity have promoted intensive research into nanoscale alternatives.^13–15^ Gadolinium oxide (Gd_2_O_3_) NPs are particularly attractive due to their high density of paramagnetic centers and efficient water coodination, resulting in enhanced *r*_1_ values compared with molecular chelates.^16–18^ Moreover, the high atomic number of Gd (*Z* = 64) contributes to X-ray attenuation, although its relatively high cost restricts the widespread use of Gd_2_O_3_ for CT applications.^19,20^

Among alternative NPs for CT, bismuth (Bi) nanomaterials have recently emerged as promising CT contrast agents owing to their high atomic number (*Z* = 83), strong X-ray attenuation, cost-effectiveness, biocompatibility, and low toxicity.^21–24^ Bi_2_O_3_ nanostructures and Bi-containing hybrids (*e.g.*, ION@Bi_2_S_3_, Bi_2_S_3_, Bi_2_O_3_–Au) have been explored for CT or multimodal imaging, often demonstrating superior attenuation compared with iodine-based agents.^1,25–27^ Nevertheless, most Bi-based probes lack intrinsic MRI contrast capability, requiring further modification or hybridization.

Recent studies have attempted to integrate Gd and Bi into composite nanostructures to achieve dual MRI/CT contrast.^8,28–32^ However, these systems generally involve multi-step syntheses, large particle sizes (>20 nm), or insufficient colloidal stability, which hinder efficient *in vivo* imaging.^8,28–30^ Moreover, very few reports have demonstrated ultrasmall (<10 nm) Gd–Bi oxide nanocomposites with high relaxivity and robust CT attenuation, combined with surface coatings that ensure stability and biocompatibility.^10^ This knowledge gap highlights the urgent need for rationally designed Gd–Bi hybrid nanoplatforms for precision imaging.

Surface modification plays a critical role in tuning NP dispersibility, stability, and biointeractions. Polyethyleneimine (PEI), a cationic polymer with abundant amino groups, has been widely applied for NPs functionalsation.^33–35^ PEI not only enhances aqueous dispersibility and colloidal stability but also offers reactive sites for bioconjugation, enabling targeted imaging. Furthermore, its instrinsic electrostatic interaction with cell membranes may contribute to anticancer effects, provinding an additional therapeutic advantage.^36,37^

In this study, we developed a facile, wet-chemistry approach to synthesis ultra-small Bi_2_O_3_/Gd_2_O_3_ NPs coated with PEI (BGO@PEI). Compared with conventional methods, our approach enables rapid one-step synthesis under mild conditions and using inexpensive precursors, making it scalable and environmentally friendly. The resulting nanocomposites integrate the high *r*_1_-enhanced capability of Gd_2_O_3_ (16.52 mM^−1^ s^−1^, surpassing clinical agents) with the superior X-ray attenuation of Bi_2_O_3_, yielding a robust dual-mode platform for MRI and CT imaging.

## Experimental

### Chemicals

All chemicals were of high purity and used as received without further purification. Bismuth nitrate pentahydrate (Bi(NO_3_)_3˙_5H_2_O, 98%), gadolinium(iii) chloride hydrate (GdCl_3˙_*x*H_2_O, 99.99%), ethylene glycol (EG, 99.8%), polyethyleneimine (PEI, *M*_W_ = 25 000), ammonia (25–28% NH_3_ in water solution) and absolute ethanol were purchased from Sigma-Aldrich, Ltd. Double-distilled water was throughout the experiments.

### Synthesis of BGO@PEI NPs

Ultrasmall BGO coated with PEI NPs were synthesized *via* one-pot modified polyol method. In a typical synthesis, 5 mmol of Bi(NO_3_)_3˙_5H_2_O was dissolved in 40 mL of EG in a 100 mL three-necked flask containing 4 g of PEI under magnetic stirring until fully dissolved. The reaction mixture was heated to 80 °C, and NH_3_ solution was then added to adjust the pH to 10, maintaining this condition for 1 hour. Subsequently, 5 mmol of GdCl_3_ dissolved in 20 mL EG was added, and the reaction was continued for an additional 6 hours under continuous N_2_ atmosphere. After cooling to room temperature, the BGO@PEI was collected by centrifugation and washed four times with distilled water and absolute ethanol to remove residual reagents, and finally redispersed in distilled water at a concentration of 10 mg mL^−1^.

### Characterisation

The particle size and morphology of BGO@PEI NPs were study by transmission electron microscopy (TEM, JEM1010, JEOL) and high-resolution transmission electron microscopy (HRTEM, JEM-2100, JEOL). Selected area electron diffraction (SAED) and X-ray diffraction (XRD, Cu Kα radiation) were used to analyze crystallinity and phase composition. Elemental composition and distribution were evaluated using energy-dispersive X-ray spectroscopy (EDS) mapping acquired on a Hitachi S-4800 FESEM, as well as HRTEM-EDX elemental mapping. The Bi and Gd contents were quantified by inductively coupled plasma mass spectrometry (ICP-MS, Agilent). X-ray photoelectron spectroscopy (XPS, Thermo Fisher, Al Kα source) was carried out to determine the oxidation states and surface composition.

Fourier-transform infrared spectroscopy (FTIR, Nicolet 6700) and thermogravimetric analysis (TGA, Netzsch, Germany) were performed to confirm PEI coating and evaluate polymer content. Dynamic light scattering (DLS) and zeta potential analysis were performed to measure hydrodynamic size distribution and colloidal stability. UV-Vis absorption spectra were recorded to evaluate electronic transitions and estimate bandgap energies.

### Cell viability measurement

The cytotoxicity of BGO@PEI NPs was evaluated by the methyl thiazolyl tetrazolium (MTT) assay using Hep-G2 (human hepatocellular carcinoma) and Vero (monkey kidney epithelial) as model cells. Cells were seeded in 96-well plates at a density of 1.5 × 10^5^ cells per well and cultured in Dulbecco's Modified Eagle's Medium (DMEM) supplemented with 10% fetal bovine serum (FBS) and 1% penicillin/streptomycin at 37 °C in a 5% CO_2_ atmosphere for 24 hours. The medium was then replaced with fresh medium containing BGO@PEI NPs at concentrations of 12.5, 25, 50, 100, and 200 µg per mL, followed by 48 hours incubation. Subsequently, 20 µL of MTT solution was added to each well and incubated for 4 hours. The resulting formazan crystals were dissolved with dimethyl sulfoxide (DMSO, Sigma-Aldrich), and optical absorbance was measured at 540 nm using a Tecan Spark microplate reader (Männedorf, Switzerland).

### MRI relaxivity measurement

MRI phantom experiments were carried out on a 1.5 T clinical scanner (Siemens Magnetom, Germany). Aqueous solutions of BGO@PEI NPs with various Gd concentrations (0, 0.031, 0.0625, 0.125, 0.25, and 0.5 mM) were prepared. *T*_1_-weighted images were acquired using a turbo spin-echo sequence with variable repetition times (TR = 100–1000 ms) and fixed echo time (TE = 12 ms), field of view (FOV) was set to 190 × 190 mm^2^, and the matrix size was 256 × 192. The longitudinal relaxivity (*r*_1_) was determined from the slope of plot of 1/*T*_1_*versus* Gd concentration.

### CT phantom imaging

CT imaging was performed using a 128-slice Somatom Perspective scanner (Siemens, Germany) at source voltage of 80–130 kV. Aqueous dispersions of BGO@PEI NPs with different total metal (Bi + Gd) concentrations (1.25, 2.5, 5.0, 7.5, and 10 mM) were embbed in 2% agarose. X-ray attenuation was quantified in Hounsfield units (HU) using region-of-interest (ROI) analysis with eFilm software (Merge Healthcare, Chicago, IL, USA).

### *In vivo* MRI/CT imaging

All animal experiments were conducted in accordance with national regulations and the institutional guidelines of Vinh International Hospital, Vietnam. Tumor-bearing mice (≈50 g) were anesthetised by intraperitoneal (IP) injection of Zoletil 50 (10–25 mg kg^−1^). A PBS dispersion of BGO@PEI NPs was subsequently administered *via* IP injection. After imaging, animals were allowed to recover from anesthesia and were returned to cages with free access to food and water.

### *In vivo* MRI

MRI studies were performed on the same 1.5 T clinical scanner as used for phantom studies. Each mouse received 300 µL of BGO@PEI NPs dispersion ([Gd] = 9.7 mM) *via* IP injection. *T*_1_-weighted images were collected at before injection, and at 10 min, 30 min, and 60 min post-injection, using the following parameters: TR/TE = 400/11 ms, matrix size = 256 × 256, FOV = 400 × 400 mm^2^, slice thickness = 3 mm, and number of averages = 2. Relative contrast enhancement at each time point was quantified based on signal intensity changes:
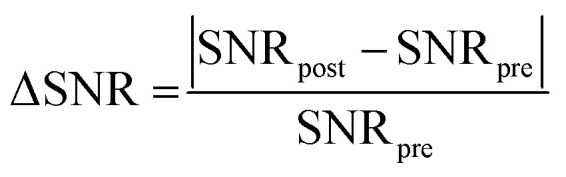


### *In vivo* CT

CT scans were performed on the same 128-slice scanner as described above using the following parameters: tube voltage = 80 kVp (31 mA s), slice thickness = 1 mm, FOV = 278 × 295 mm^2^, matrix size = 541 × 510, and number of averages = 2. CT images were obtained before and after IP injection of 0.8 mL BGO@PEI NPs dispersion in PBS ([Bi + Gd] = 26.4 mM). Image reconstruction and analysis were carried out using Syngo CT VC30 software (easyIQ version).

## Results and discussion

### Mophology and structural characterisation

**Fig. 1:**
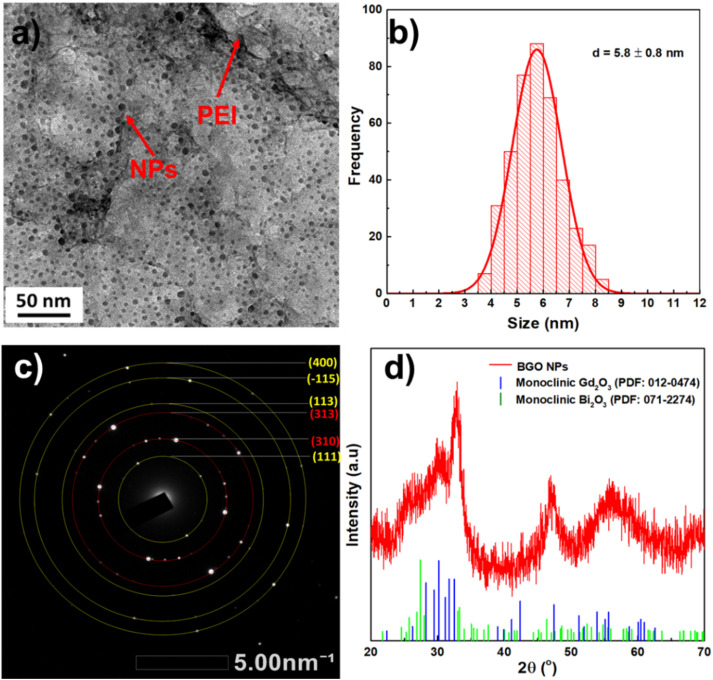
(a) TEM image, (b) size distribution, (c) SEAD pattern and (d) XRD pattern of BGO@PEI NPs.

The morphology of BGO@PEI NPs was determined by transmission electron microscopy analysis (TEM), as shown in Fig. 1a and b. The TEM image reveals that the NPs exhibit a spherical shape with an average size of 5.8 ± 0.8 nm. The uniform morphology indicates good control over the crystallization process under the one-step polyol synthesis conditions. Additionally, high-resolution TEM (HRTEM) analysis (Fig. S1) further confirmed the presence of well-defined lattice fringes, while the corresponding fast Fourier transform (FFT) pattern (inset) exhibited distinct diffraction spots that were assigned to the (111) plane of monoclinic Bi_2_O_3_ and the (310) plane of monoclinic Gd_2_O_3_, respectively, thereby corroborating the polycrystalline nature of the NPs and supporting the coexistence of both oxide phases at the nanoscale.

To further confirm the phases present, selected area electron diffraction (SAED) analysis was conducted, as shown in Fig. 1c. SAED pattern displayed concentric rings with spots, indicating that the as-synthesized BGO@PEI NPs are polycrystalline. Interplanar spacings, calculated from the diffraction pattern, are consistent with those reported for monoclinic phase Gd_2_O_3_ (PDF No. 012-0474) and monoclinic Bi_2_O_3_ (PDF No. 071-2274). The diffraction rings correspond to the Bragg reflections of (111), (113), (−115), and (400) planes of Bi_2_O_3_ (highlighted in yellow) and the (310) and (313) planes of Gd_2_O_3_ (highlighted in red).

The crystalline phases of the sample were also examined by XRD, and the corresponding patterns are presented in Fig. 1d. The observed diffraction peaks are in good agreement with the standard reference data for both monoclinic Gd_2_O_3_ (PDF No. 012-0474) and Bi_2_O_3_ (PDF No. 071-2274), confirming the coexistence of both oxide phases. In addition, the peaks are significantly broader than those reported for the pristine materials, which can be attributed to the ultrafine particle size and associated microstrain within the oxide domains, partial overlap of Bi_2_O_3_ and Gd_2_O_3_ reflections,^38^ and the presence of a relatively thick and amorphous PEI coating on the NPs. These combined effects account for the noisy background and the difficulty in resolving certain expected peaks.

**Fig. 2:**
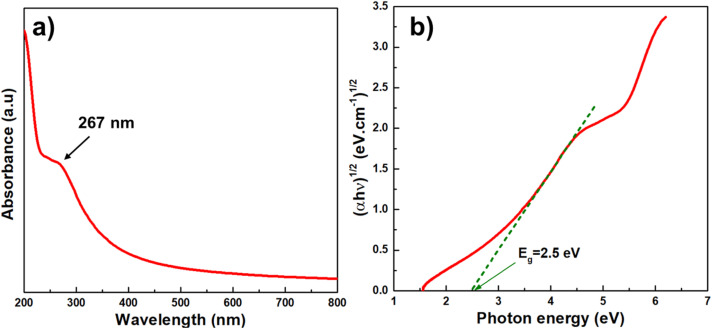
(a) UV-Vis spectrum and (b) optical band gap energy calculation by Tauc plot method of BGO@PEI NPs.

The optical properties of BGO@PEI NPs were investigated using UV-Vis absorption spectrum. As shown in Fig. 2a, the absorption spectrum of the NPs exhibits a strong absorption band in the UV region with a distinct shoulder at approximately 267 nm, corresponding to metal–oxygen (M–O) charge-transfer transitions involving Bi–O and Gd–O bonds in the oxide. The presence of this absorption band further confirms the semiconductor nature of the NPs.

The optical bandgap energy (*E*_g_) was estimated using the Tauc method, based on the (*αhν*)^1/2^*versus* photon energy (*hν*) plot for an indirect bandgap transition (Fig. 2b).^39^ Extrapolation yielded *E*_g_ ≈ 2.5 eV, lower than the reported bandgap of pristine Bi_2_O_3_ or Gd_2_O_3_ (∼2.7–3.0 eV).^40^ The reducing is possible attributed to interfacial interactions between the two oxide phases and the effect of PEI coating. Such synergistic coordination and structural disorder introduced by the polymer shell likely perturb the electronic states, thereby facilitating modified electronic transitions.

**Fig. 3:**
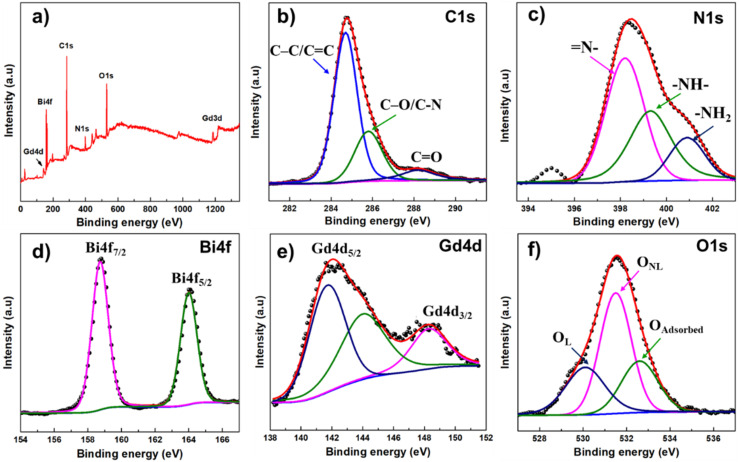
(a) Scan survey XPS spectrum of BGO@PEI NPs and high-resolution XPS spectra of: (b) C1s, (c) N1s, (d) Bi4f, (e) Gd4d and (f) O1s.

The oxidation state and surface chemical composition of BGO@PEI NPs are investigated by X-ray photoelectron spectroscopy (XPS). The survey spectrum (Fig. 3a) confirms the presence of Bi, Gd, O, N and C elements, consistent with the precursor composition. High-resolution XPS spectra provided further insight into the chemical states. The C1s spectrum (Fig. 3b) was deconvoluted into three components at 284.7, 285.8 and 288.2 eV, assigned to C–C/C=C, C–O/C–N and C=O bondings, respectively. The N1s spectrum (Fig. 3c) exhibited peaks at 398.2, 399.3, and 400.8 eV, corresponding to N–, –NH–, and –NH_2_ group, confirming successful PEI coating. The Bi4f spectrum (Fig. 3d) displayed two prominent peaks at 158.7 and 164 eV, attributed to the Bi 4f_7/2_ and Bi 4f_5/2_, with a spin–orbit separation of 5.3 eV, characteristic of Bi^3+^.^41^ No metalic Bi was detected in the sample. The Gd 4d spectrum (Fig. 3e) showed two main peaks at 142.1 and 148.3 eV, corresponding to Gd 4d_5/2_ and Gd 4d_3/2_, indicative of Gd^3+^.^42,43^ A satellite peak at 144.1 eV rose from multiplet splitting due to interactions between the Gd 4d core levels and partially filled 4f orbitals, further confirming that the intrinsic electronic structure of Gd^3+^ was retained in the nanocomposites.^44,45^

The O1s spectrum (Fig. 3f) resolved into three peaks. The signal at 530.1 eV was assigned to lattice oxygen (O_L_), corresponding to O^2−^ ions strongly bound to Bi^3+^ and Gd^3+^ within the stable oxide framework. The dominant peak at 531.5 eV corresponded to non-lattice oxygen species (O_NL_) at defect sites or with incomplete coordination. The higher binding energies peak at 532.6 eV was attributed to adsorbed oxygen, including hydroxyl groups, molecular water, or surface O_2_.

**Fig. 4:**
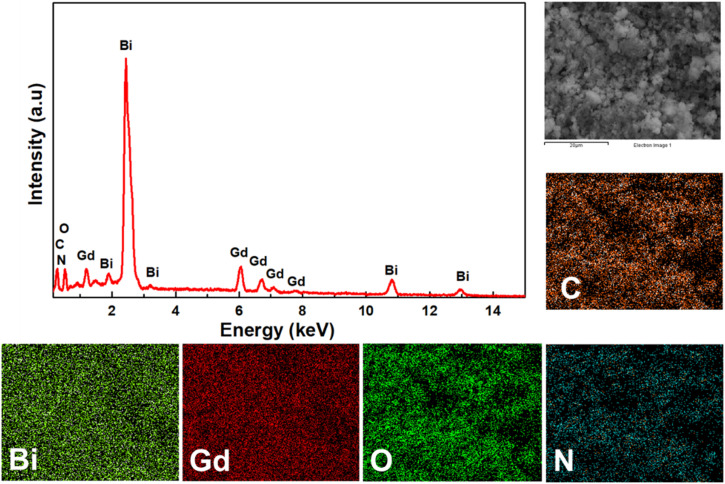
EDX spectrum and EDX elemental mapping of BGO@PEI NPs.

Energy-dispersive X-ray spectroscopy (EDS) was used for qualitatively and semi-quantitatively elemental analysis of BGO@PEI NPs. The EDS spectrum (Fig. 4) confirmed the presence of Bi, Gd, and O as the three major constituents, while additional C and N signals envidenced the presence of the PEI coating. SEM-EDS elemental mapping further reveals a uniform distribution of Bi, Gd, and O across the NPs, demonstrating the compositional homogeneity of the material. To further explore the local elemental distribution, HRTEM-EDS mapping was performed over a 200 nm region, as shown in Fig. S2. Although not at single particle resolution, this mapping revealed overlapping Bi, Gd, and O signals, with a relatively stronger Gd signal alongside Bi in the mapped region. The apparent Gd enrichment likely arises from the probing regions near the NP surface, consistent with the potential formation of a Gd-rich shell. To obtain precise quantification, inductively coupled plasma mass spectrometry (ICP-MS) was conducted. The measured Bi/Gd molar ratio was 1.73, higher than the theoretical precursor ratio of 1, suggesting a lower incorporation efficiency of Gd compared with Bi under the given synthesis conditions. Collectively, these observations are consistent with the formation of Bi_2_O_3_/Gd_2_O_3_ composite NPs with a possible core/shell-like distribution.

**Fig. 5:**
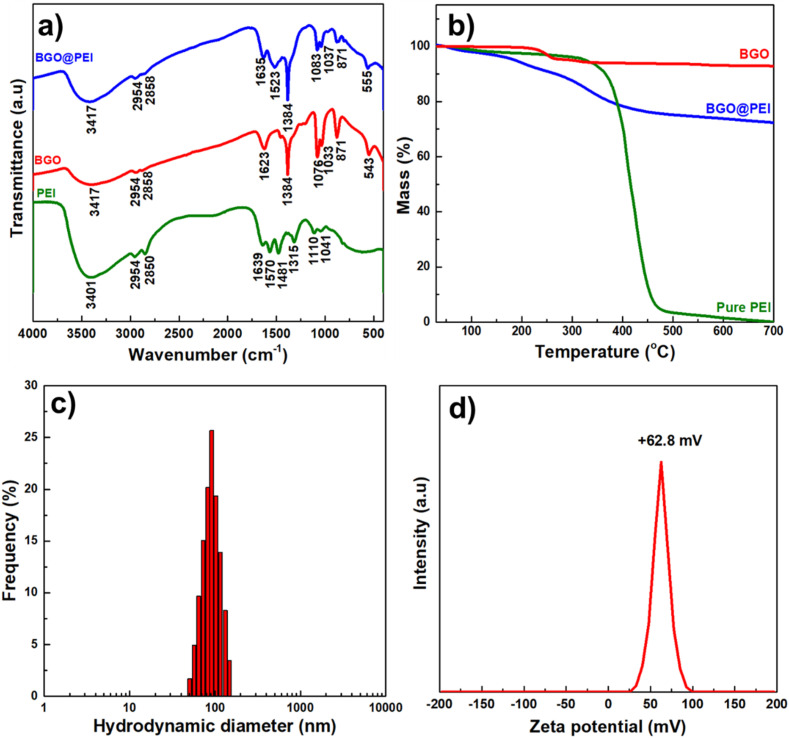
(a) FTIR spectra, (b) TGA plot, (c) DLS spectrum and (d) zeta potential of BGO@PEI NPs.

FTIR spectroscopy was employed to verify the successful coating of PEI on BGO NPs. Fig. 5a presents the FTIR spectra of pure PEI, bare BGO NPs, and BGO@PEI NPs.

For pure PEI, a broad absorption band at 3200–3450 cm^−1^ is observed, corresponding to the stretching vibrations of N–H bonds in amine groups, overlapped with O–H stretching from adsorbed water. Peaks at 2954 and 2850 cm^−1^ are assigned to C–H stretching vibrations of the hydrocarbon chains. The bands at 1643, 1570, and 1481 cm^−1^ are assigned to N–H bending vibrations of primary and secondary amines, while the additional peaks at 1315, 1110, and 1041 cm^−1^ confirm the presence of C–N stretching vibrations.^46^

In the spectrum of BGO@PEI NPs, characteristic absorption bands of both PEI and BGO are observed, indicating the coexistence of both components. The absorption bands at 3417, 2954, and 2858 cm^−1^ (N–H and C–H vibrations) and the signals at 1635 and 1523 cm^−1^ (N–H bending vibrations) validate the successful attachment of the polymer layer onto the BGO surface. Meanwhile, the absorption peaks at 871 cm^−1^ and 1041 cm^−1^ correspond to the stretching vibrations and the bending vibrations of Bi–O bonds,^38,47^ the absorption peak at 555 cm^−1^ is attributed to Gd–O lattice vibrations,^38^ demonstrating that the oxide framework of BGO remains intact after surface modification.

TGA analysis was performed to estimate the PEI content on the NPs (Fig. 5b). Pure PEI exhibits a major weight loss between 300–450 °C and nearly complete decomposition above 550 °C, consistent with the thermal instability of organic polymers. In contrast, bare BGO NPs show negligible weight loss throughout 250–700 °C, reflecting its high thermal stability. For BGO@PEI NPs, three weight-loss stages were observed: (i) below 150 °C (2.6%), due to adsorbed water and residual solvents; (ii) 150–250 °C (7.1%), corresponding to decomposition of EG; and (iii) from 250–550 °C (18.3%) attributed to PEI degradation. These results indicate that the inorganic oxide core accounts for approximately 72% of the total mass.

Dynamic light scattering (DLS) analysis (Fig. 5c) revealed a narrow size distribution without secondary peaks, indicating good dispersion stability in aqueous solution. The average hydrodynamic diameter of the NPs (∼91.6 nm) is significantly larger than the particle size observed by TEM. This difference arises because DLS measures nanoparticles in their fully hydrated and dynamic state, reflecting not only the inorganic BGO cores but also the expanded PEI shell, the surrounding solvated counterion layer, and possibly transient, reversible soft aggregates that may form in solution, whereas TEM only measures the individual inorganic BGO cores, consistent with the previous reports.^48^ Zeta potential analysis (Fig. 5d) showed a relatively high positive value of +62.8 mV, indicative of strong electrostatic repulsion between particles, which effectively prevents aggregation and ensures long-term colloidal stability in suspension.

To further assess the robustness of BGO@PEI NPs under physiologically relevant conditions, the colloidal behavior was examined in media of varying ionic strengths and pH values. As shown in Fig. S3a and b, the NPs remained uniformly dispersed without visible sedimentation after 24 h in NaCl concentrations up to 350 mM and across a wide pH range (pH 1–11), with precipitation occurring only under extreme conditions (380 mM or pH 13). The corresponding DLS profiles (Fig. S3c and d) further support these observations, showing that the hydrodynamic diameter remained essentially unchanged (∼90–100 nm) within these ranges, with noticeable size enlargement and peak broadening only at 380 mM NaCl or pH 13, consistent with partial aggregation in these extreme environments. Notably, the DLS peaks shifted slightly toward smaller and narrower size distributions in strongly acidic media (pH 1–4), suggesting enhanced electrostatic stabilization due to increased protonation of the PEI shell at these pH values.

Long-term storage stability was also confirmed by zeta potential measurements (Fig. S3e), which showed only a slight decrease from +62.8 to +55.7 mV after more than 18 months of storage, as well as by the unchanged visual appearance of the stored sample. In addition, the strong and continuous Tyndall scattering observed in Fig. S3f provides qualitative evidence that the NPs remain well dispersed without forming large aggregates. These results demonstrate the excellent colloidal stability of BGO@PEI NPs, supporting their suitability for biomedical imaging applications.

### Cytotoxicity

**Fig. 6:**
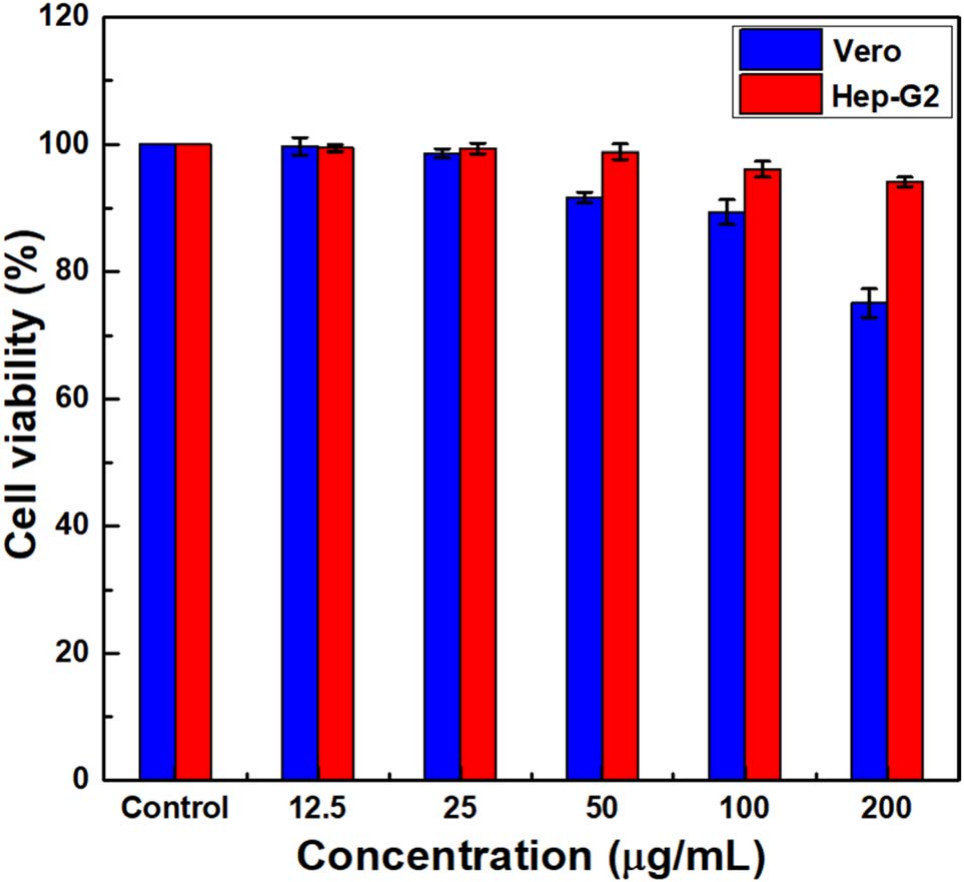
*In vitro* viability of Vero and Hep-G2 cells after incubation with various concentrations of BGO@PEI NPs for 48 hours.

Evaluation of cellular toxicity is essential to determine the biosafety of nanomaterials prior to *in vivo* applications. In this study, the *in vitro* cytotoxicity of BGO@PEI NPs was examined using the MTT assay on normal (Vero) and cancer (Hep-G2) cell lines (Fig. 6). Cells were incubated with BGO@PEI NP suspensions at different concentrations (0, 12.5, 25, 50, 100, and 200 µg mL^−1^) for 48 hours and viability was determined by quantifying formazan absorbance. The results revealed that Hep-G2 cells maintained high viability (∼94%) even at 200 µg mL^−1^, indicating good biocompatibility.

In contrast, Vero cells exhibited reduced viability (∼75%) under the same conditions, suggesting a more pronounced effect on normal cells after prolonged exposure. This difference in cellular response may stem from variations in metabolic activity, NP uptake efficiency, and oxidative stress responses between the two cell types.^49–51^ Overall, BGO@PEI NPs exhibited low cytotoxicity *in vitro* supporting their suitability for biomedical imaging. Nonetheless, cellular responses can vary depending on the cell type, exposure duration, and physicochemical characteristics of the nanomaterials. These findings provide an essential foundation for subsequent *in vivo* toxicity assessments, thereby supporting the potential of BGO@PEI NPs for biomedical imaging and therapeutic applications.

### MRI/CT phantom images

**Fig. 7:**
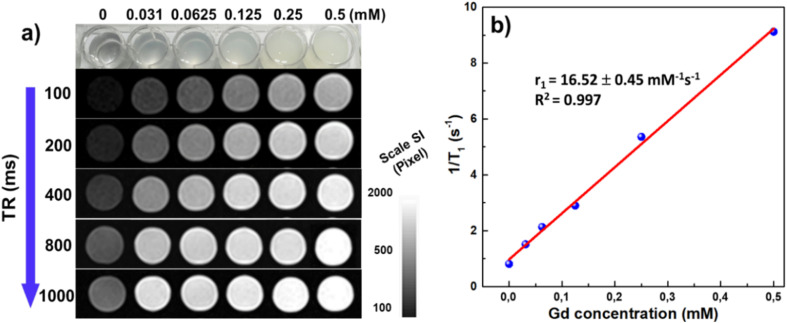
(a) *T*_1_-weighted MR imaging and (b) plots of 1/*T*_1_*versus* Gd concentrations for BGO@PEI NPs. The slopes of the 1/*T*_1_ plot represent the longitudinal relaxivity (*r*_1_).

The *T*_1_-weighted (*T*_1_W) MRI performance of BGO@PEI NPs was evaluated on a clinical 1.5 T MRI scanner using aqueous dispersions with Gd concentrations ranging from 0 to 0.5 mM. As shown in Fig. 7a, image brightness increased progressively with Gd concentration, confirming concentration-dependent signal enhancement. Quantitative analysis (Fig. 7b) revealed a longitudinal relaxivity (*r*_1_) of 16.52 mM^−1^ s^−1^, nearly four times higher than that of the clinical Gd-chelate contrast agents (Gd-DTPA, typically *r*_1_ ≈ 4–5 mM^−1^ s^−1^ at 1.5 T).^10,52^ Such a high relaxivity indicates that BGO@PEI NPs can generate strong MRI contrast even at relatively low doses, undercoring their potential as efficien *T*_1_ agents.

The *T*_1_ contrast enhancement mechanism primarily originates from the interaction between Gd^3+^ ions within the Gd_2_O_3_ lattice and surrounding water protons.^8^ The ultrasmall particle size (around 5.8 nm) results in a larger surface-to-volume ratio, exposing more Gd^3+^ sites to water molecules and facilitating inner-sphere relaxation. When water protons approach the vicinity of Gd^3+^ ions, their rotational motion is modulated at the Larmor frequency, facilitating energy transfer from the protons to the lattice and thereby shortening the longitudinal relaxation time (*T*_1_).^53,54^ This process contributes directly to signal amplification in *T*_1_W images. Additionally, the PEI coating provides colloidal stability and prevents aggregation, ensuring effective nanoparticle dispersion in aqueous media and maximizing the available Gd–water interactions. Together, these features contribute to efficient *T*_1_ shortening and pronounced image brightening in MRI.

**Fig. 8:**
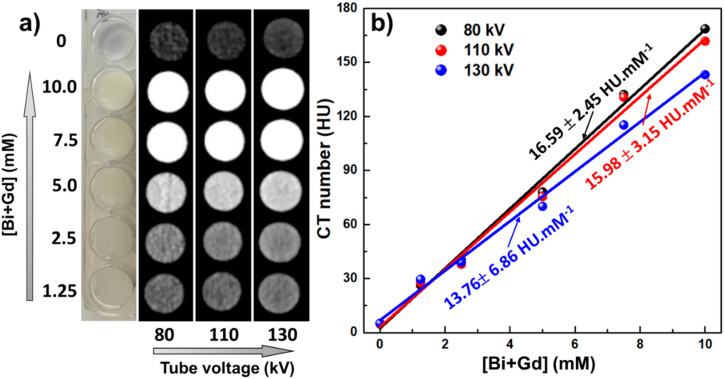
(a) CT images and (b) plots of X-ray attenuation (HU) of BGO@PEI NPs as a function of the Bi + Gd concentration.

The simultaneous presence of two high-*Z* elements, bismuth (Bi, *Z* = 83) and gadolinium (Gd, *Z* = 64), endows the BGO@PEI NPs with strong X-ray attenuation, making them promising CT contrast agents. In this study, *in vitro* CT imaging were performed on a 128-slice CT scanner using aqueous dispersions of BGO@PEI NPs with total metal (Bi + Gd) concentrations of 1.25 to 10 mM. As shown in Fig. 8a, image brightness increased progressively with concentrations. Quantitative analysis (Fig. 8b) yielded a linear correlation between CT values and concentration, with a slope of 16.59 ± 2.45 HU mM^−1^ at 80 kV, approximately 3–4 times higher than that of the commercial iodine-based contrast agent.^20,55^ These results indicate the superior CT contrast performance of the NPs.

This enhancement is primarily attributed to Bi, which has a much higher atomic number and X-ray attenuation coefficient than iodine (*Z* = 53), the standard element in current CT contrast agents. Gd (*Z* = 64) also contributes, both by enhancing MRI *T*_1_ contrast and by providing additional attenuation at lower X-ray energies. The synergistic combination of Gd_2_O_3_ and Bi_2_O_3_ within a single nanostructure therefore enables more efficient X-ray attenuation compared with previously reported hybrid systems, such as Gd_2_O_3_–iodine composite or Au NPs conjugated with Gd chelates.^56,57^ Notably, compared to our previously developed Bi/Bi_2_O_3_@PAA NPs,^58^ the BGO@PEI NPs demonstrated even higher attenuation efficiency, highlighting the effectiveness of the dual-component design strategy.

The attenuation efficiency was further evaluated under varying tube voltages of 80, 110, and 130 kV. As shown in Fig. 8b, the corresponding slopes were 16.59, 15.98, and 13.76 HU mM^−1^, respectively. The slight decrease at higher voltage indicates that the CT contrast enhancement capability diminishes slightly at higher X-ray energies. This trend can be explained by the interaction characteristics of high-*Z* elements with X-rays. At 80 kVp, which is close to the K-edge absorption energy of Bi (90.5 keV), photoelectric absorption dominates, leading to stronger X-ray attenuation and higher CT signal intensities. In contrast, at 110 and 130 kV, the contribution of photoelectric absorption is progressively replaced by Compton scattering, resulting in reduced CT signal intensity. Furthermore, the presence of Gd (*Z* = 64), with a K-edge of 50.2 keV, also helps rationalize this behavior: as the tube voltage increases to 130 kV, the X-ray spectrum shifts to higher energies far beyond the Gd K-edge, thereby reducing its photoelectric contribution and diminishing its role in the overall CT signal.

Importantly, BGO@PEI NPs maintained relatively stable X-ray attenuation across the entire tested voltage range, with only minor reductions at higher energies. This robustness suggests that the material can perform effectively under diverse CT imaging conditions, including low-voltage protocols, commonly employed for soft tissue and vascular imaging as well as high-voltage or low-dose CT scans, typically used for bone imaging. This wide-range effectiveness highlights BGO@PEI NPs as a promising candidate for clinical CT imaging applications.

The *r*_1_ relaxivity and X-ray attenuation efficiency of BGO@PEI NPs were systematically compared with those of previously reported Bi/Gd-based NPs. As summarized in Table S1, both parameters are comparable to or exceed those of most Bi–Gd nanosystems described in the literature.

The enhanced *r*_1_ relaxivity and X-ray attenuation of BGO@PEI NPs can be attributed to their ultrasmall solid-state structure, which ensures a high density of exposed Gd^3+^ and Bi^3+^ ions. This architecture facilitates efficient inner-sphere water coordination to Gd^3+^, thereby boosting longitudinal relaxivity, while the high Bi^3+^ content and compact Bi_2_O_3_/Gd_2_O_3_ domains maximize photoelectric absorption for CT imaging. Moreover, the hydrophilic PEI coating establishes a highly hydrated surface environment, promoting rapid water exchange at Gd^3+^ sites and further contributing to *r*_1_ enhancement. These findings indicate that the unique structural features of BGO@PEI NPs, including ultrasmall particle size, solid Bi_2_O_3_/Gd_2_O_3_ core domains, and high surface accessibility of metal ions, are critical factors underlying their superior dual-modal MRI/CT imaging performance.

### *In vivo* CT and MRI images

**Fig. 9:**
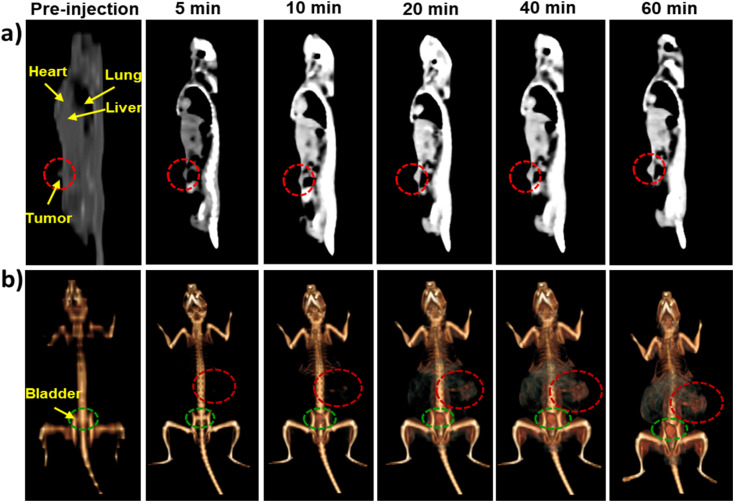
CT images of Sarcoma 180 tumor-bearing mice: pre- and post-treatment by IP injection of the BGO@PEI NPs: (a) sagittal section, (b) 3D renderings whole-body.

After confirming the excellent X-ray attenuation efficiency, favorable *T*_1_-weighted MRI contrast enhancement, and good biocompatibility of BGO@PEI NPs *in vitro*, their potential as *in vivo* CT/MRI contrast agents was further evaluated. *In vivo* CT scans were performed before and after IP injection of BGO@PEI NPs dispersed in PBS into Sarcoma 180 tumor-bearing mice. As shown in Fig. 9a, following NP administration, the major organs (heart, lung, liver, and bladder) and tumor region became increasingly distinguishable. A noticeable enhancement at the tumor site appeared as early as 10 min post-injection, with the strongest contrast observed at 60 min. 3D rendering images further confirmed the clear visualization of the tumor (Fig. 9b).

**Fig. 10:**
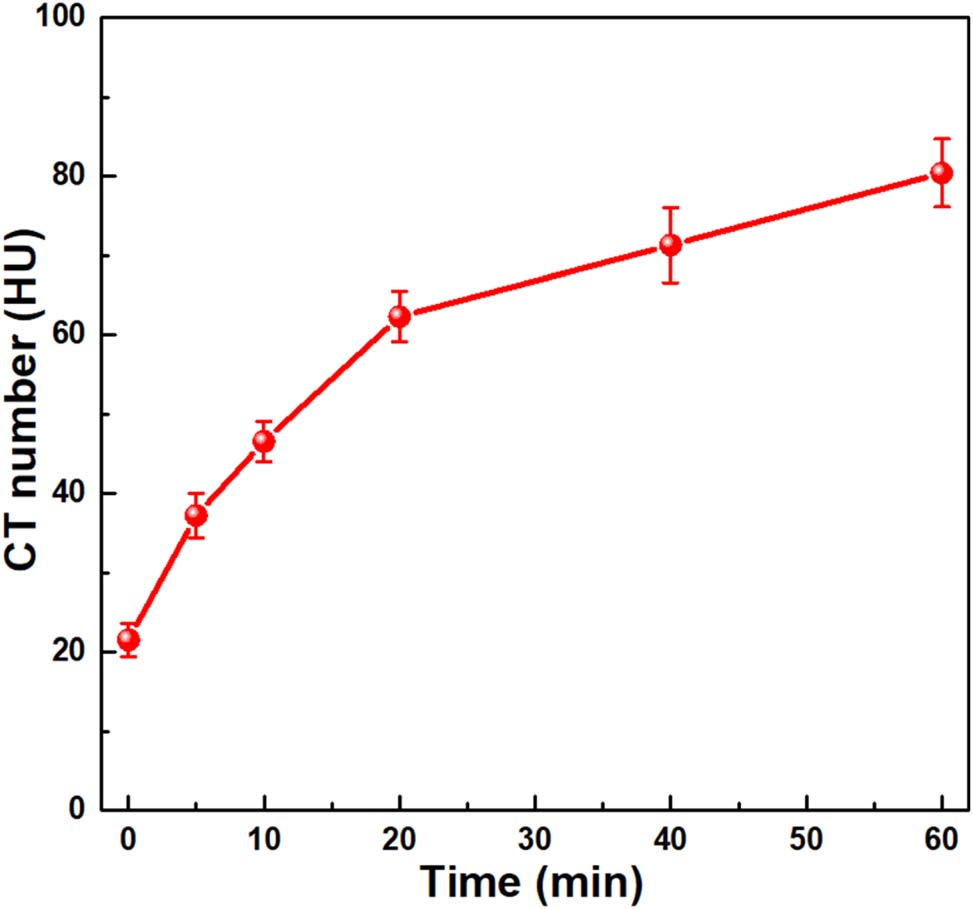
Plots of CT values as a function of time (*n* = 2).

Analysis of the i*n vivo* CT imaging (Fig. 10) revealed that the CT signal intensity at the tumor site markedly increased from 21.5 HU (pre-injection) to 80.4 HU at 60 min post-injection, corresponding to nearly a fourfold enhancement. This pronounced signal elevation indicates efficient tumor accumulation *via* the enhanced permeability and retention (EPR) effect. The pronounced ΔHU values can be ascribed to the synergistic contributions of the Bi_2_O_3_/Gd_2_O_3_ composite structure and PEI coating that facilitated improved intratumoral penetration and prolonged retention.

In addition to tumor accumulation, CT images also displayed strong signal enhancement in the bladder region (Fig. 9b), suggesting partial renal excretion of the nanoparticles. The bright bladder signal is consistent with the clearance of ultrasmall, hydrophilic fractions of BGO@PEI NPs through glomerular filtration. These findings further support the potential BGO@PEI NPs as CT contrast agents.

**Fig. 11:**
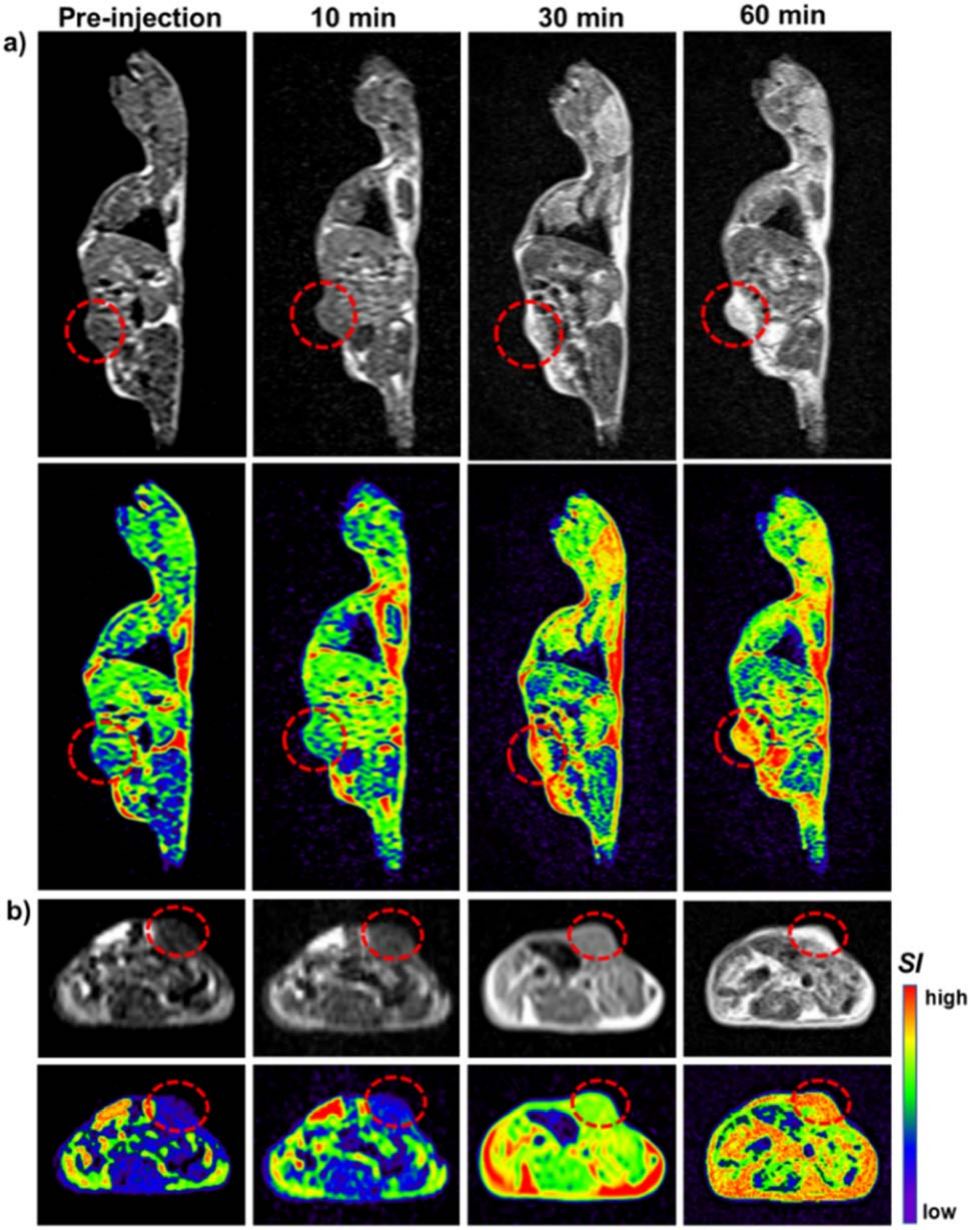
T_1_W-MRI images of Sarcoma 180 tumor-bearing mice: pre- and post-treatment by intraperitoneal injection of BGO@PEI NPs: (a) sagittal section, (b) axial section of the tumor region.

**Fig. 12:**
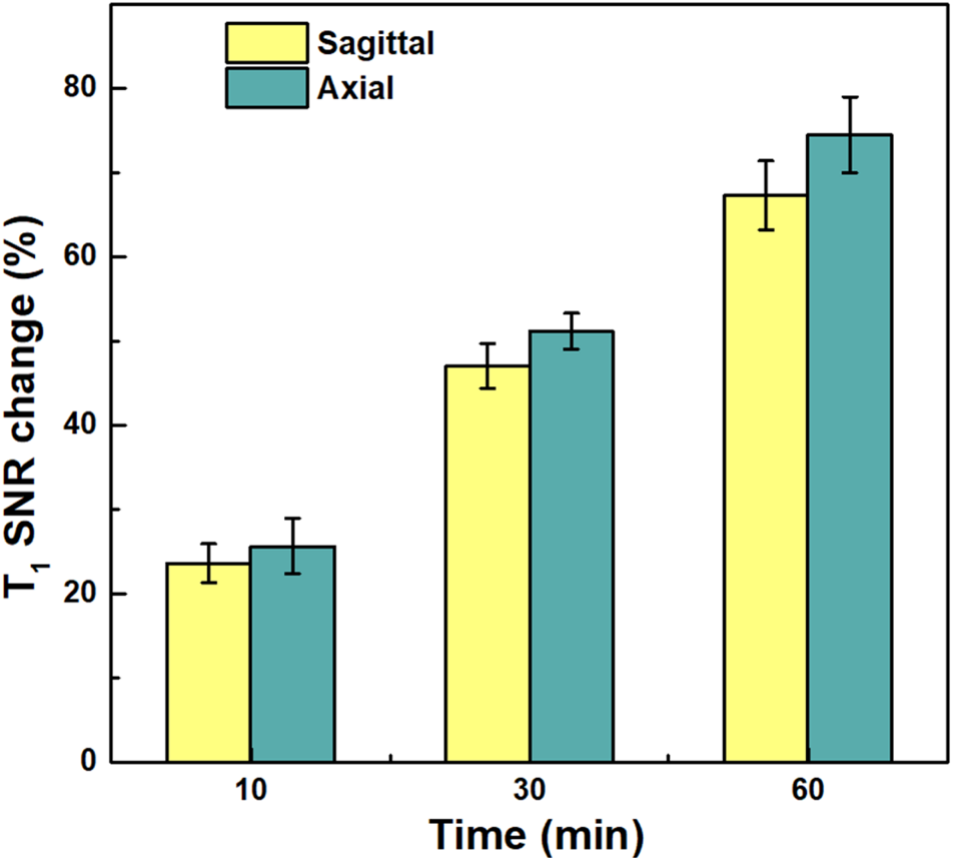
SNR changes in tumor of *T*_1_-weighted images after administration (*n* = 2).

In parallel, *in vivo T*_1_-weighted MRI demonstrated a marked increase in tumor signal intensity over time (Fig. 11). At 60 min post-injection, the ΔSNR reached 67% in sagittal slices and 75% in axial slices (Fig. 12). This pronounced *T*_1_ brightening reflects not only effective NP accumulation at the tumor site but also the strong *T*_1_-shortening ability of Gd^3+^ ions within the nanostructure.

Overall, these results demonstrate that BGO@PEI NPs functions as an effective dual-modal MRI/CT contrast agent, enabling clear imaging at relatively low concentrations, exhibiting high X-ray attenuation, and providing sustained tumor signal enhancement suitable for non-invasive diagnostics. The integration of two high-*Z* elements (Bi and Gd) together with PEI coating conferred synergistic benefits in contrast performance and colloidal stability, highlighting their potential for multimodal imaging and tumor progression monitoring.

## Conclusions

In summary, we have successfully synthesized PEI-coated bismuth–gadolinium oxide composite nanoparticles (BGO@PEI NPs) *via* a facile one-step polyol route. The resulting nanomaterial integrates the high X-ray attenuation capability of Bi with the strong *T*_1_ MRI contrast enhancement of Gd, while the PEI coating ensures excellent water dispersibility, colloidal stability, and favorable biocompatibility. Both *in vitro* and *in vivo* evaluations demonstrated good biosafety and efficient dual-modal MRI/CT imaging performance, with significantly improved longitudinal relaxivity and CT attenuation compared to clinical contrast agents. The obtained results demonstrated BGO@PEI NPs as a promising multifunctional nanoplatform for next-generation diagnostic imaging, offering valuable potential for integration into personalised and precision medicine.

## Author contributions

Le T. T. Tam conceptualized the study, designed the methodology, performed data analysis, and wrote the original draft of the manuscript. Le V. Thanh, Le G. Nam, and Le T. Tam performed the MRI and CT measurements and contributed to data validation. Doan T. Tung and Hoang T. Dung conducted the experiments and curated the associated data. Ho D. Quang carried out the toxicity tests and collected the associated data. Ngo T. Dung provided technical assistance during the laboratory work and supported data acquisition. Le T. Lu acquired funding, contributed to manuscript revision, and supervised the overall project. All authors reviewed and approved the final version of the manuscript.

## Conflicts of interest

There are no conflicts to declare.

## Supplementary Material

RA-015-D5RA07455J-s001

## Data Availability

All data supporting the findings of this study are available within the article and its supplementary information (SI). Supplementary information is available. See DOI: https://doi.org/10.1039/d5ra07455j.
