# Supplementary material for: Facile synthesis of polyethylenimine coated Bi2O3/Gd2O3 composite nanoparticles as multimodal MRI/CT contrast agents
Source: RSC Adv. 2025 Dec 8;15(56):48393–404. doi: 10.1039/d5ra07455j (PMC12683713; doi:10.1039/d5ra07455j)
Supplement: RA-015-D5RA07455J-s001 [file RA-015-D5RA07455J-s001.pdf]

Supplementary Information (SI) for RSC Advances.
This journal is © The Royal Society of Chemistry 2025

Supporting Information

Facile synthesis of Polyethylenimine coated Bi_2O_3/Gd_2O_3 composite nanoparticles as multimodal MRI/CT contrast agents

Le T. T. Tam[a,b], Le V. Thanh[c], Le G. Nam[c], Doan T. Tung[a], Hoang T. Dung[a,b], Le T. Tam[d], Ho D. Quang[d], Ngo T. Dung[a] and Le T. Lu[a,b*]

[a]*Institute of Materials Science, Vietnam Academy of Science and Technology, 18 Hoang Quoc Viet, Hanoi, Vietnam.*

[b]*Graduate University of Science and Technology, Vietnam Academy of Science and Technology, 18 Hoang Quoc Viet, Hanoi, Vietnam.*

[c]*Hoan My Vinh Hospital, 99 Pham Đinh Toai, Vinh City, Vietnam.*

[d]*Vinh University, 182 Le Duan, Vinh City, Vietnam.*

*Corresponding author: lult@ims.vast.ac.vn

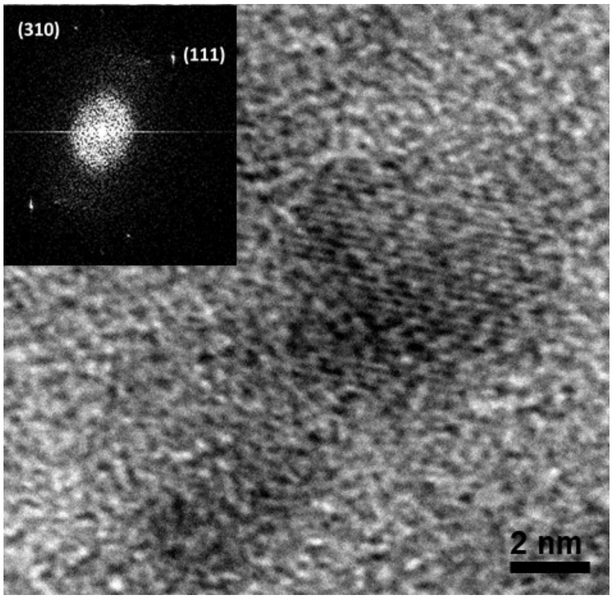

Fig. S1. HRTEM image with an inset FFT image of BGO@PEI NPs.

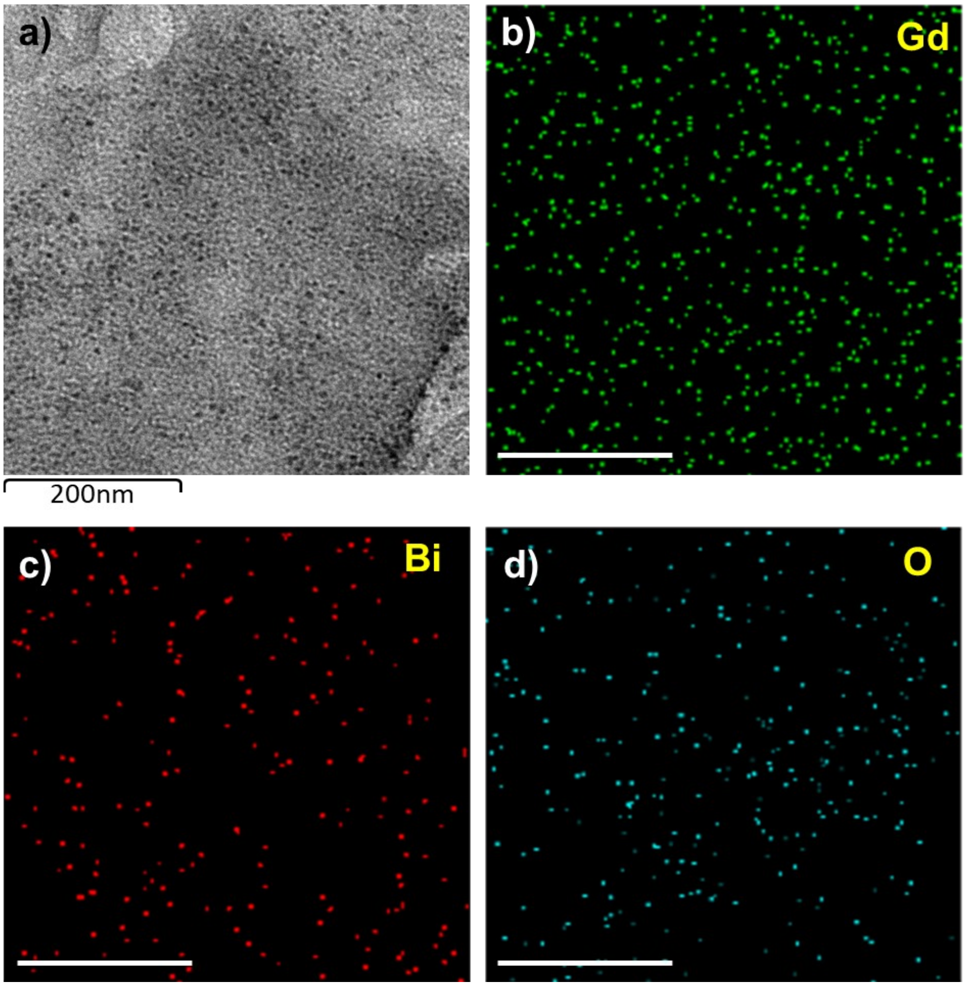

Fig. S2. a) HRTEM-EDS elemental mapping of BGO@PEI NPs.

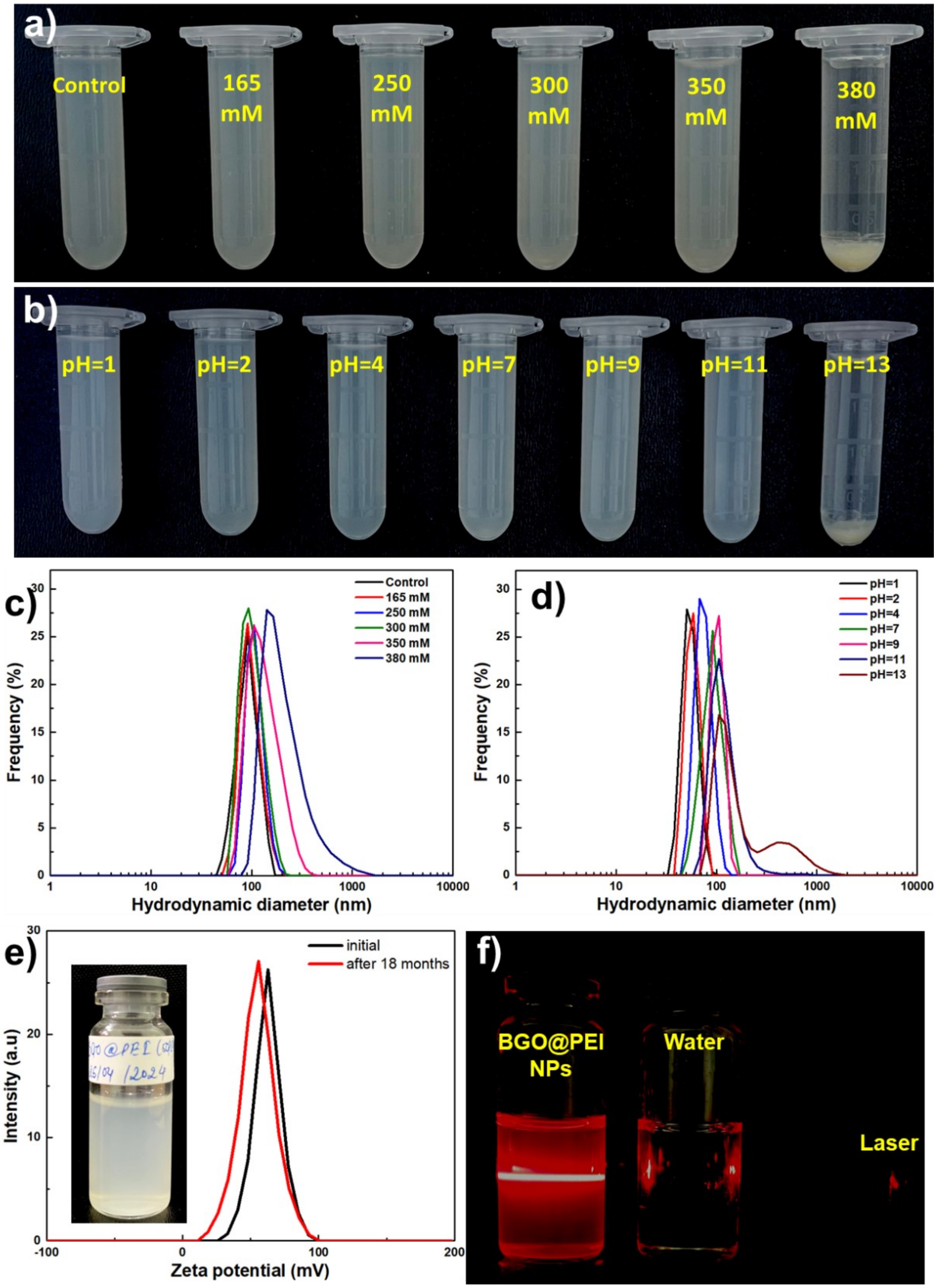

Fig. S3. Photographs and DLS spectra of BGO@PEI NP dispersions at various pH values (a, c) and NaCl concentrations (b, d) after 24 h; (e) Zeta potential measurements together with a photograph of transparent BGO@PEI NP dispersions stored undisturbed in aqueous media for over 18 months since synthesis, confirming their long-term colloidal stability; (f) Photograph illustrating the Tyndall effect (laser light scattering) of the NP colloids.

Table S1. Comparison of r_1 value and X-ray attenuation efficiency of BGO@PEI NPs with other studies.

Contrast agent	Morphology, Size (nm)	X-ray attenuation efficiency (HU.mM^{-1})	r_1 value (mM^{-1}s^{-1})	References
**BGO@PEI NPs**	**Spherical, 5.8**	**16.59 (80 kV) 15.78 (110 kV) 13.76 (130 kV)**	**16.52 (1.5T)**	**This work**
**$Bi_{1.5}Gd_{0.5}O_3$@PAA**	Spherical, 1.8	16.3 (35 kV), 15.9 (50 kV), 14.1 (75 kV)	35.8 (3 T)	[1]
**SiBiGdNP**	-	4.26 (45 kV)	4.87 (7 T)	[2]
**Gd-PEG-Bi NPs**	Spherical, 10	8.04 (80 kV)	4.9 (3 T)	[3]
**Bi/Gd-doped C QDs**	Spherical, 2.8	3.43	4.29 (7 T)	[4]
**Gd-DTPA (Magnevist)**	NA		4.7 (3T)	[3]
**Ultravist**	NA	4.4 (70 kV)		[5]
**Iohexol**	NA	5.6 (80 kV)		[3]
**Iobitridol**	NA	3.85 (120 kV)		[6]

[1] D. Zhao, A. Baek, T. Tegafaw, Y. Liu, S. Liu, H. Yue, E. Mulugeta, J. Yang, J. A. Park, H. Lee, D. W. Hwang, S. Kim, J. Kim, Y. Chang, and G. H. Lee, *ACS Appl. Nano Mater.*, 2025, **8**, 13663-13675.

[2] A. Detappe, E. Thomas, M. W. Tibbitt, S. Kunjachan, O. Zavidij, N. Parnandi, E. Reznichenko, F. Lux, O. Tillement and R. Berbeco, *Nano Letters*, 2017, **17**, 1733–1740.

[3] B. Wu, S. T. Lu, H. Yu, R. F. Liao, H. Li, B. V. L. Zafitatsimo, Y. S. Li, Y. Zhang, X. L. Zhu, H. G. Liu, H. B. Xu, S.-W. Huang and Z. Cheng, *Biomaterials*, 2018, **159**, 37−47.

[4] Q. Meng, Y. Wang, C. Li and X. Hu, *New J. Chem.*, 2022, **46**(35), 16970-16980.

[5] A. Ghazanfari, S. Marasini, X. Miao, J. A. Park, K. H. Jung, M. Y. Ahmad, H. Yue, S. L. Ho, S. Liu, Y. J. Jang, K. S. Chae, Y. Chang and G. H. Lee, *Colloids Surf. A*, 2019, **576**(5), 73–81.

[6] P. Lei, R. An, P. Zhang, S. Yao, S. Song, L. Dong, X. Xu, K. Du, J. Feng and H. Zhang, *Adv. Funct. Mater.*, 2017, 1702018.
